# A bulk form Cu-based ferromagnetic semiconductor (La,Ba)(Cu,Mn)SO with the Curie temperature up to 170 K

**DOI:** 10.1038/s41598-023-41895-8

**Published:** 2023-09-05

**Authors:** Jinou Dong, Cui Ding, Xueqin Zhao, Lingfeng Xie, Qiaolin Yang, Xun Pan, Guoxiang Zhi, Licheng Fu, Yilun Gu, Fanlong Ning

**Affiliations:** 1 Zhejiang Province Key Laboratory of Quantum Technology and Device and School of Physics, Zhejiang University, Hangzhou, 310027 China; 2 Collaborative Innovation Center of Advanced Microstructures, Nanjing University, Nanjing, 210093 China; 3 State Key Laboratory of Silicon and Advanced Semiconductor Materials, Zhejiang University, Hangzhou, 310027 China; 4 Science and Technology Innovation Center, Chifeng High-Tech Industrial Development Zone, Chifeng, 025250 China

**Keywords:** Condensed-matter physics, Ferromagnetism, Magnetic properties and materials, Semiconductors, Spintronics

## Abstract

We report the ferromagnetism in a new bulk form Cu-based magnetic semiconductor (La,Ba)(Cu,Mn)SO, which is iso-structural to the prototypical iron-based 1111-type superconductor LaFeAsO. Starting from the parent compound LaCuSO, carriers are introduced via the substitutions of La for Ba while spins are introduced via the substitutions of Cu for Mn. Spins are mediated by carriers, which develops into the long range ferromagnetic ordering. The maximum Curie temperature $T_{C}$ reaches up to $\sim$ 170 K with the doping levels of 10% Ba and 5% Mn. By comparing to the (La,Sr)(Cu,Mn)SO where Sr and Mn are co-doped into LaCuSO, we demonstrate that negative chemical pressure would suppress the ferromagnetic ordering.

## Introduction

The discovery of ferromagnetism in III-V semiconductors doped with magnetic atom doping has opened a new window in the research of spintronics^1–5^. It has been proposed that the Curie temperature $T_{C}$ of (Ga,Mn)As would be raised to room temperature when spin and carrier densities are optimized^6^. Unfortunately, due to the mismatch of valence between Mn$^{2+}$ and Ga$^{3+}$, the solid solubility is severely limited, hindering more Mn atoms doped in (Ga,Mn)As. As of today, $T_{C}$ in (Ga,Mn)As has a maximum value of $\sim$ 200 K with Mn doping level up to $\sim$ 12%^7–10^. On the other hand, the non-equivalent substitutions of Mn for Ga in (Ga,Mn)As introduce magnetic moments and carriers simultaneously, which makes it difficult to separate spins and charges, and investigate their individual contributions to the formation of ferromagnetism. Besides, (Ga,Mn)As is fabricated in the form of thin-film by molecular beam epitaxy (MBE) that is extremely sensitive to the preparation process, which makes some research inconclusive^11–13^. Hence, to understand the general mechanism of ferromagnetic ordering and seek for magnetic semiconductors (MSs) with $T_{C}$ close to room temperature are still the forefront research in spintronics.

Recently, many novel bulk MSs that are the structural derivatives of iron-based superconductors have emerged, such as 111-type Li(Zn,Mn)As ($T_{C}$ = 50 K)^14^, Li(Zn,Mn)P ($T_{C}$ = 34 K)^15^, Li(Zn,Cr)As ($T_{C}$ = 218 K)^16^, 122-type (Ba,K)(Zn,Mn)$_{2}$As$_{2}$ ($T_{C}$ = 230 K)^17, 18^, N-type Ba(Zn,Co)$_{2}$As$_{2}$ ($T_{C}$ = 45 K)^19^ and 1111-type (La,Ba)(Zn,Mn)AsO ($T_{C}$ = 40 K)^20^, which are iso-structural to the iron-based superconductors 111-type LiFeAs^21^, 122-type (Ba,K)Fe$_{2}$As$_{2}$^22^ and 1111-type LaFeAsO$_{1-\delta }$^23^, respectively. In these bulk MSs, it has been demonstrated that the long range ferromagnetic ordering is arising from the doped magnetic atoms, and the mechanism of ferromagnetism is the same as that of (Ga,Mn)As^18, 20, 24^. Furthermore, these bulk MSs have the advantages of decoupled spin and carrier doping, which enables the concentrations of charges and spins to be tuned separately. Thirdly, the bulk form specimens enable the application of magnetic techniques at microscopic level, such as nuclear magnetic resonance (NMR)^25^, muon spin relaxation ($\mu$SR) and neutron scattering^26, 27^.

In order to improve $T_{C}$, researchers have examined the influence of physical and chemical pressures on MSs. As expected, the effects of pressure on MSs are quite different. It has been shown that the applied physical pressure, positive chemical pressure and negative chemical pressure all suppress the $T_{C}$ in (Ba,K)(Zn,Mn)$_{2}$As$_{2}$^28, 29^. On the other hand, the positive chemical pressure would enhance the Curie temperature $T_{C}$ by 18% in Ba(Zn,Co)$_{2}$As$_{2}$^30^ and 30% in (La,Ca)(Zn,Mn)AsO^31^. In addition, the bulk MSs mentioned above are all Zn-based MSs. Apparently, in order to shed light on the origin of ferromagnetism, we also need to pay more attention to other type MSs, such as Cu-based MSs. In past, a new Cu-based MS^32^ has been reported with $T_{C}$ around 200 K when Sr and Mn were co-doped into the parent compound LaCuSO, which is a wide band gap (3.1 eV) semiconductor^33, 34^. It is natural to wonder what would happen if Ba (which has larger ionic radius than Sr$^{2+}$) and Mn are co-doped into LaCuSO, and how chemical pressure would affect the ferromagnetism in 1111-type Cu-based MSs.

In this paper, we report the successful synthesis of a 1111-type bulk MS (La,Ba)(Cu,Mn)SO. Spins are introduced via the substitutions of Cu for Mn, and carriers are introduced via the substitutions of La for Ba into the parent compound LaCuSO. The resultant maximum $T_{C}$ is $\sim$ 170 K with Ba and Mn doping up to 10% and 5%, respectively. Basing on the experimental results, we unequivocally demonstrate that carriers play important roles in the formation of the ferromagnetism. Comparing to (La,Sr)(Cu,Mn)SO, the Weiss temperature $\theta$ of (La,Ba)(Cu,Mn)SO with the same doping levels is about 37% lower, which is apparently due to the reason that the ionic radius of Ba$^{2+}$ is larger than that of Sr$^{2+}$, that is, negative chemical pressure suppresses the ferromagnetic ordering in (La,AE)(Cu,Mn)SO (AE=Sr, Ba) MS systems.

## Results

### X-ray diffraction and transport

**Figure 1:**
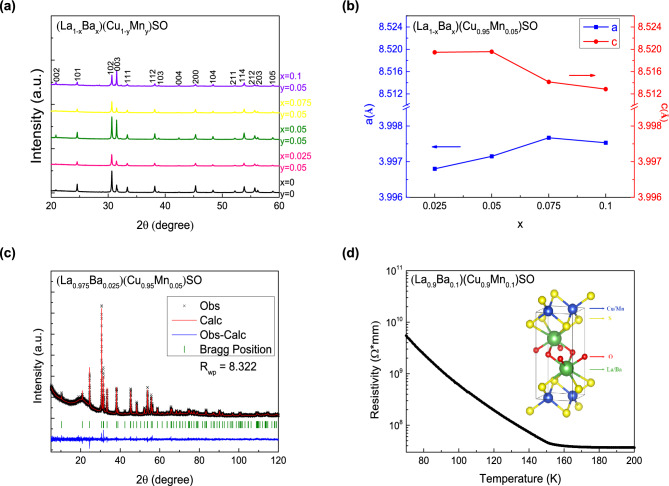
(**a**) The X-ray diffraction patterns for (La$_{1-x}$Ba$_{x}$)(Cu$_{1-y}$Mn$_{y}$)SO ($0\le x\le 0.1$, $0 \le y \le 0.05$) with (hkl) index. (**b**) The lattice parameters $\textit{a}$ and $\textit{c}$ of (La$_{1-x}$Ba$_{x}$)(Cu$_{0.95}$Mn$_{0.05}$)SO ($0 \le x \le 0.1$). (**c**) The Rietveld refinement of (La$_{0.975}$Ba$_{0.025}$)(Cu$_{0.95}$Mn$_{0.05}$)SO. (**d**) The electrical resistivity of the (La$_{0.9}$Ba$_{0.1}$)(Cu$_{0.9}$Mn$_{0.1}$)SO on a log scale. Inset is the plot of the crystal structure of LaCuSO.

We show the crystal structure and x-ray diffraction patterns of the samples in Fig. 1. The Bragg peaks in Fig. 1a can be well indexed by a tetragonal ZrCuSiAs-type structure with *P*4/*nmm* (No.129) space group, indicating that both parent compound LaCuSO and doped compounds are iso-structural to the 1111-type iron-based superconductor LaFeAsO^23, 34^. In Fig. 1b, we plot the lattice parameters of the specimens obtained by Rietveld refinement. Comparing with the parent compound LaCuSO, all doping samples have larger lattice parameters. No impurity phase has been observed. We show the Rietveld refinement profile of (La$_{0.975}$Ba$_{0.025}$)(Cu$_{0.95}$Mn$_{0.05}$)SO in Fig. 1c by using the GSAS-II package as an example, and the resulting weighted reliability factor $R_{wp}$ is $\sim$ 8.3%, implying a well and reliable refinement^35^. In Fig. 1d, we show the electrical resistivity of the (La$_{0.9}$Ba$_{0.1}$)(Cu$_{0.9}$Mn$_{0.1}$)SO down to 70 K (It increases beyond the measurement limitation below $\sim$ 70 K). We can see that the resistivity curve retains its semiconducting behavior. According to the Arrhenius equation *R* = $R_{0}$*exp($E_{g}$/($k_{B}$*T)), we obtained the activation energy $E_{g}$ $\sim$ 0.14 eV which is much smaller than 3.1 eV of the parent semiconductor LaCuSO.

**Figure 2:**
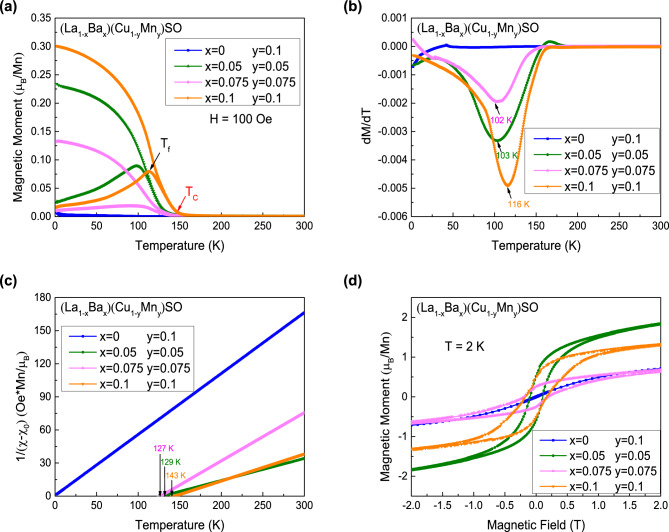
(**a**) Temperature dependent magnetization for (La$_{1-x}$Ba$_{x}$)(Cu$_{1-y}$Mn$_{y}$)SO ($0 \le x \le 0.1$, $0.05 \le y \le 0.1$) measured in zero field cooling (ZFC) and field cooling (FC) condition under 100 Oe external field. The red arrow marks the Curie temperature ($T_C$) and the black arrow marks the freezing temperature ($T_f$) for (La$_{0.9}$Ba$_{0.1}$)(Cu$_{0.9}$Mn$_{0.1}$)SO, respectively. (**b**) The *dM*/*dT* versus *T* curves for (La$_{1-x}$Ba$_{x}$)(Cu$_{1-y}$Mn$_{y}$)SO ($0 \le x \le 0.1$, $0.05 \le y \le 0.1$). The arrows mark the temperature *T*(*dM*/*dT*) for samples. (**c**) The reverse of $\chi -\chi _{0}$ versus temperature for (La$_{1-x}$Ba$_{x}$)(Cu$_{1-y}$Mn$_{y}$)SO ($0 \le x \le 0.1$, $0.05 \le y \le 0.1$). The arrows mark the Weiss temperature ($\theta$) for samples. (**d**) The iso-thermal magnetic hysteresis measurement for (La$_{1-x}$Ba$_{x}$)(Cu$_{1-y}$Mn$_{y}$)SO ($0 \le x \le 0.1$, $0.05 \le y \le 0.1$) under 2 K.

### Magnetic properties of (La$_{1-x}$Ba$_{x}$)(Cu$_{1-y}$Mn$_{y}$)SO ($0 \le x \le 0.1$, $0.05 \le y \le 0.1$)

In Fig. 2, we show the results of DC magnetic properties of (La$_{1-x}$Ba$_{x}$)(Cu$_{1-y}$Mn$_{y}$)SO ($0 \le x \le 0.1$, $0.05 \le y \le 0.1$). We plot the temperature dependence of the magnetization under field cooling (FC) and zero field cooling (ZFC) conditions with an applied external field of 100 Oe in Fig. 2a. In the measured temperature range, no anomaly or transition can be observed in La(Cu$_{0.9}$Mn$_{0.1}$)SO, demonstrating that doping magnetic Mn atoms alone into LaCuSO can not result in any type of magnetic ordering. However, as marked by the red arrow for $x=0.1$ and $y=0.1$ sample, there is a sharp increase of the DC magnetization M in the temperature range of 140–150 K when both carriers and local magnetic moments are provided. The sudden sharp increase of DC magnetization is indicative of a ferromagnetic transition, which is denoted as the Curie temperature ($T_{C}$). $T_{C}$ is $\sim$ 142 K for the doping levels of $x=0.1$ and $y=0.1$. In this case, (La,Ba) substitutions introduce the holes while (Cu,Mn) substitutions introduce the local magnetic moments, respectively. Hence, spins can be mediated by carriers, which will lead to the long range ferromagnetic ordering. It is consistent with Zener‘s model which is considered to explain the origin of ferromagnetism in (Ga,Mn)As system^6^. In Zener‘s model, the holes can mediate local moments arising from Mn spins through RKKY-like interaction effectively. Furthermore, we can also see the bifurcation between FC and ZFC curves around 110 K. The temperature of the bifurcation is denoted as T$_{f}$, which is marked by the black arrow as shown in Fig. 2a, indicating the freezing temperature of individual spins or domain wall motion.

**Figure 3:**
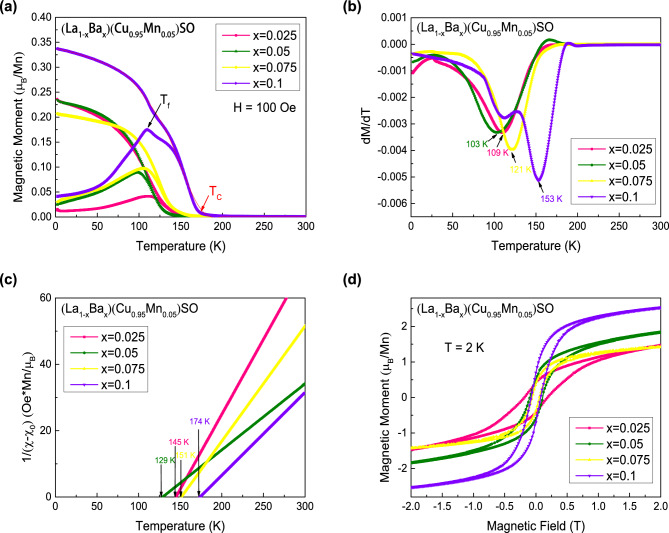
(**a**) Temperature dependent magnetization for (La$_{1-x}$Ba$_{x}$)(Cu$_{0.95}$Mn$_{0.05}$)SO ($0 \le x \le 0.1$) measured in zero field cooling (ZFC) and field cooling (FC) condition under 100 Oe external field. The red arrow marks the Curie temperature ($T_C$) and the black arrow marks the freezing temperature ($T_f$) for $x=0.1$, respectively. (**b**) The *dM*/*dT* versus *T* curves for (La$_{1-x}$Ba$_{x}$)(Cu$_{0.95}$Mn$_{0.05}$)SO ($0 \le x \le 0.1$). The arrows mark the temperature *T*(*dM*/*dT*) for samples. (**c**) The reverse of $\chi -\chi _0$ versus temperature for (La$_{1-x}$Ba$_{x}$)(Cu$_{0.95}$Mn$_{0.05}$)SO ($0 \le x \le 0.1$). The arrows mark the Weiss temperature ($\theta$) for samples. (**d**) The iso-thermal magnetic hysteresis measurement for (La$_{1-x}$Ba$_{x}$)(Cu$_{0.95}$Mn$_{0.05}$)SO ($0 \le x \le 0.1$) under 2 K.

We plot the first derivative of magnetization versus temperature in Fig. 2b. Here, we focus on the temperature of the extreme point of *dM*/*dT*, which is denoted as *T*(*dM*/*dT*). We find that the change of *T*(*dM*/*dT*) with dopings is consistent with that of $T_{C}$. Theoretically, to accurately determine the Curie temperature $T_{C}$ in a ferromagnetic compound, the method of Arrot plot can be used to define $T_{C}$ explicitly^36^. That is, the iso-thermal magnetization data points ($M^{2}$ versus *H*/*M*) around $T_{C}$ in high magnetic field would fall on a series of parallel lines. The line corresponding $T_{C}$ would pass through the origin. Practically, we found this method would also bring some uncertainties. Instead, we found that *T*(*dM*/*dT*) is proportional to $T_{C}$, and using *T*(*dM*/*dT*) is very efficient and accurate to compare the variation of $T_{C}$ for different samples. We also plot the inverse of DC susceptibility versus temperature in Fig. 2c. We can see that the curve of La(Cu$_{0.9}$Mn$_{0.1}$)SO passes through the origin, which indicates that there is no magnetic transition. According to the Curie-Weiss law $\chi$ = $\chi _{0}$ + *C*/(*T*-$\theta$), the *x*-axis intercept of the linear fitting curves is defined as the Weiss temperature $\theta$, where $\chi _{0}$ is the temperature-independent component and *C* is the Curie constant. $\theta$ values are marked by vertical arrows in Fig. 2c, the variation trend of $\theta$ is consistent with that of $T_{C}$ as well. Moreover, the positive $\theta$ values also indicate the ferromagnetic interaction between Mn-ions. Additionally, we found our “$T_{C}$” values are larger than *T*(*dM*/*dT*), but smaller than the Weiss temperature $\theta$ (which is strictly determined by the intercept of $1/\chi$ versus *T*). We can also obtain the effective magnetic moment $\mu _{eff}$ from the formula *C* = $N\mu _{0}\mu _{eff}^{2}/3K_{B}$. The resultant $\mu _{eff}$ are in the order of magnitudes $\sim$ 5 ${\mu }_B/Mn$, which are much larger than those of spin glass ($\sim$ 0.01 ${\mu }_B/Mn$) but close to 5.9 ${\mu }_B/Mn$, as expected for fully magnetic individual Mn$^{2+}$ moments^37, 38^. In Fig. 2d, we plot the iso-thermal magnetization measured at 2 K. Similarly, ferromagnetic hysteresis loops can be observed for all polycrystals except La(Cu$_{0.9}$Mn$_{0.1}$)SO. The obtained data for the Curie temperature $T_C$, the temperature *T*(*dM*/*dT*), the Weiss temperature $\theta$, the effective moment $\mu _{eff}$, and the coercive field $H_c$ are tabulated in Table 1.

### Magnetic properties of (La$_{1-x}$Ba$_{x}$)(Cu$_{0.95}$Mn$_{0.05}$)SO ($0 \le x \le 0.1$)

Next, we investigate the influence of carriers on the ferromagnetic ordering by fixing the Mn concentration at $y=0.05$. In Fig. 3, we display the results of DC magnetic properties of (La$_{1-x}$Ba$_{x}$)(Cu$_{0.95}$Mn$_{0.05}$)SO ($0 \le x \le 0.1$). The temperature dependence of the magnetization under FC and ZFC conditions in an applied magnetic field of 100 Oe is shown in Fig. 3a. We can find a significant increase of M in the temperature range of 120–180 K for all curves. Interestingly, the more carrier doping levels, the higher $T_{C}$. $T_{C}$ reaches to the maximum value of $\sim$ 170 K with Ba doping up to 10%.

Similarly, the first derivative of magnetization versus temperature and the inverse of DC susceptibility versus temperature are plotted in Fig. 3b and c, respectively. The variation trend of *T*(*dM*/*dT*) and $\theta$ are the same as that of the $T_{C}$. It‘s worth noting that the effective moments are determined to be 3–5 ${\mu }_B/Mn$ under this circumstance, indicating that Mn-ion is in the high spin state *S* = $\frac{5}{2}$ with the valence of $+2$. $\mu _{eff}$ are all less than the expected value of 5.9 ${\mu }_B/Mn$, which are similar to the situation in (Ga,Mn)As system^39, 40^. In general, the effective moments are affected by the valence of Mn ions, and the indirect interaction arising from carriers mediated ferromagnetism and the direct antiferromagnetic interaction of Mn atoms at nearest-neighbor sites. When we calculate the effective moments, we suppose all doped Mn atoms are involved into the magnetic interaction. While small amount of diluted Mn atoms may not effectively interact with carriers but they were counted in. This may be another reason that the effective magnetic moments are much smaller than the 5.9 ${\mu }_B/Mn$ expected for *S* = $\frac{5}{2}$. We also display the iso-thermal magnetization at 2 K in Fig. 3d. All specimens show clear hysteresis loops, suggesting that the ferromagnetic transition has fully developed. However, the variations of coercive field and saturation moment in Figs. 2d and 3d are not monotonous with the increasing doping, which might be due to the result of competition between ferromagnetic coupling via the RKKY-like interaction and the nearest-neighbor antiferromagnetic coupling via direct exchange interaction. Likewise, the obtained data for the Curie temperature $T_C$, the temperature *T*(*dM*/*dT*), the Weiss temperature $\theta$, the effective moment $\mu _{eff}$, and the coercive field $H_c$ are tabulated in Table 1.

**Table 1:** The Curie temperature $T_C$, the temperature *T*(*dM*/*dT*), the Weiss temperature $\theta$, the effective moment $\mu _{eff}$ and the coercive field $H_c$ for (La$_{1-x}$Ba$_{x}$)(Cu$_{1-y}$Mn$_{y}$)SO ($0 \le x \le 0.1$, $0.05 \le y \le 0.1$).

*x*; *y*	$T_C$ (K)	*T*(*dM*/*dT*) (K)	$\theta$ (K)	$\mu _{eff}$ (${\mu }_B/Mn$)	$H_c$ (Oe)
0; 0.1	–	–	–	2.5	–
0.075; 0.075	121	102	127	3.2	1910
0.1; 0.1	142	116	143	4.3	1807
0.025; 0.05	131	109	145	3.1	1808
0.05; 0.05	126	103	129	4.7	1005
0.075; 0.05	140	121	151	3.6	598
0.01; 0.05	170	153	174	4.2	805

## Discussion and summary

We have successfully synthesized a new bulk form Cu-based MS (La,Ba)(Cu,Mn)SO by solid-state reaction method, which is iso-structural to the iron-based 1111-type superconductor LaFeAsO. The maximum $T_{C}$ can reach up to $\sim$ 170 K when the doping levels of Ba and Mn are 10% and 5%, respectively. These results demonstrate that the ferromagnetic transition occurs only when both carriers and spins are introduced. However, the competition between ferromagnetic coupling and antiferromagnetic coupling may have a considerable influence on the ferromagnetism. Additionally, the Weiss temperature $\theta$ is defined as the “Curie temperature $T_{C}$” in (La,Sr)(Cu,Mn)SO^32^. We now compare the Weiss temperature $\theta$ for the same doping levels of (La,Sr)(Cu,Mn)SO and (La,Ba)(Cu,Mn)SO. The $\theta$ of (La$_{0.925}$Sr$_{0.075}$)(Cu$_{0.925}$Mn$_{0.075}$)SO is $\sim$ 200 K. However, the $\theta$ of (La$_{0.925}$Ba$_{0.075}$)(Cu$_{0.925}$Mn$_{0.075}$)SO decreases by $\sim$ 37% to 127 K. This is apparently because the ionic radius of Ba$^{2+}$ (1.36 Å) is larger than that of Sr$^{2+}$ (1.18 Å), as can be seen that (La,Ba)(Cu,Mn)SO has larger bond length than that of (La,Sr)(Cu,Mn)SO. Consequently, the ferromagnetic interaction in (La,Ba)(Cu,Mn)SO is suppressed. This phenomenon has also been observed in Ba(Zn,Co)$_{2}$As$_{2}$^30^ and (La,Ca)(Zn,Mn)AsO^31^. To summarize, this new bulk MS can be used as a new reference to study the origin of ferromagnetism in magnetic semiconductors with higher $T_{C}$.

## Methods

### Material synthesis

We synthesized (La$_{1-x}$Ba$_{x}$)(Cu$_{1-y}$Mn$_{y}$)SO ($0 \le x \le 0.1$, $0 \le y \le 0.1$) polycrystalline specimens by solid-state reaction with high-purity starting materials of La, La$_{2}$O$_{3}$, MnS, CuS and BaO$_{2}$. According to the chemical formula, we mixed and slowly heated the ingredients up to $950$ °C in an evacuated silica tube, where the mixture was held for about 33 h before cooling down to the room temperature. Secondly, the products were grounded, pelleted, and sintered at $950$ °C for another 33 h again to achieve complete reaction.

### Experimental characterization

Powder x-ray diffraction was performed at room temperature using a PANalytical x-ray diffractometer (model EMPYREAN) with monochromatic Cu $K_{\alpha 1}$ radiation. The DC magnetic properties of the polycrystals were conducted by using a Quantum Design magnetic property measurement system (MPMS-3). The electrical resistivity was measured on sintered pellets by a Quantum Design physical property measurement system (PPMS).

## Data Availability

All data generated or analysed during this study are included in this published article or available from the corresponding author on reasonable request.
